# Multidrug-resistant pathogens and ventilator-associated pneumonia in critically ill COVID-19 and non-COVID-19 patients: a prospective observational monocentric comparative study

**DOI:** 10.1186/s12931-024-02779-1

**Published:** 2024-04-18

**Authors:** Giorgia Montrucchio, Eleonora Balzani, Gabriele Sales, Anna Vaninetti, Francesca Grillo, Anna Chiara Trompeo, Marinella Zanierato, Vito Fanelli, Silvia Corcione, Francesco Giuseppe De Rosa, Antonio Curtoni, Cristina Costa, Luca Brazzi

**Affiliations:** 1 Department of Surgical Sciences, University of Turin, Turin, Italy; 2 Department of Anesthesia, Intensive Care and Emergency, Città della Salute e della Scienza Hospital, Turin, Italy; 3 Department of Medical Sciences, University of Turin, Turin, Italy; 4 Tufts University School of Medicine, Boston, MA 02111 USA; 5 Department of Public Health and Paediatrics, University of Turin, Turin, Italy; 6 Microbiology and Virology Laboratory, Città Della Salute e Della Scienza Hospital, Corso Dogliotti 14, Turin, 10126 Italy

**Keywords:** COVID-19, Ventilator-acquired pneumonia, Critical care, Multidrug resistant organisms, Difficult to treat organisms, Antimicrobial resistance

## Abstract

**Background:**

The COVID-19 pandemic has increased the incidence of ventilator-associated pneumonia (VAP) among critically ill patients. However, a comparison of VAP incidence in COVID-19 and non-COVID-19 cohorts, particularly in a context with a high prevalence of multidrug-resistant (MDR) organisms, is lacking.

**Material and Methods:**

We conducted a single-center, mixed prospective and retrospective cohort study comparing COVID-19 patients admitted to the intensive care unit (ICU) of the “Città della Salute e della Scienza” University Hospital in Turin, Italy, between March 2020 and December 2021 (COVID-19 group), with a historical cohort of ICU patients admitted between June 2016 and March 2018 (NON-COVID-19 group). The primary objective was to define the incidence of VAP in both cohorts. Secondary objectives were to evaluate the microbial cause, resistance patters, risk factors and impact on 28 days, ICU and in-hospital mortality, duration of ICU stay, and duration of hospitalization).

**Results:**

We found a significantly higher incidence of VAP (51.9% - *n* = 125) among the 241 COVID-19 patients compared to that observed (31.2% - *n* = 78) among the 252 NON-COVID-19 patients. The median SOFA score was significantly lower in the COVID-19 group (9, Interquartile range, IQR: 7–11 vs. 10, IQR: 8–13, *p* < 0.001). The COVID-19 group had a higher prevalence of Gram-positive bacteria-related VAP (30% vs. 9%, *p* < 0.001), but no significant difference was observed in the prevalence of difficult-to-treat (DTR) or MDR bacteria. ICU and in-hospital mortality in the COVID-19 and NON-COVID-19 groups were 71% and 74%, vs. 33% and 43%, respectively. The presence of COVID-19 was significantly associated with an increased risk of 28-day all-cause hospital mortality (Hazard ratio, HR: 7.95, 95% Confidence Intervals, 95% CI: 3.10-20.36, *p* < 0.001). Tracheostomy and a shorter duration of mechanical ventilation were protective against 28-day mortality, while dialysis and a high SOFA score were associated with a higher risk of 28-day mortality.

**Conclusion:**

COVID-19 patients with VAP appear to have a significantly higher ICU and in-hospital mortality risk regardless of the presence of MDR and DTR pathogens. Tracheostomy and a shorter duration of mechanical ventilation appear to be associated with better outcomes.

**Supplementary Information:**

The online version contains supplementary material available at 10.1186/s12931-024-02779-1.

## Introduction

Ventilator-associated pneumonia (VAP) typically develops in patients exposed to invasive mechanical ventilation (IMV) for at least 48 h [1]. In the pre-pandemic era the incidence ranged between 5% and 40% [2], but even higher rates were reported among COVID-19 patients (48–64%) [3–6].

During the Coronavirus disease 2019 (COVID-19) pandemic, an unprecedented number of patients were admitted to ICUs due to COVID-19-related severe acute respiratory syndrome and required IMV [7]. IMV is a notable risk factor for the onset of VAP. Moreover, COVID-19 considerably increased the risk of developing VAP, due to multiple factors, such as disease and therapy associated immune-paralysis, prolonged duration of mechanical ventilation and sedation and more frequent application of prone positioning [8].

Despite the frequent use of prolonged mechanical ventilation in COVID-19 patients and several data describing the epidemiology of VAP in such patients, there is a paucity of studies comparing the impact of VAP in a pre-pandemic control population and COVID-19 population [6, 9]. There is also limited literature regarding risk factors among COVID-19 patients developing VAP [10, 11] and the use of scoring systems such as Simplified Acute Physiology Score (SAPS) II and Sequential Organ Failure Assessment (SOFA) score as prognostic factors in a population with VAP [12, 13]. The need for a pre-pandemic control population becomes even more relevant if we consider countries burdened by a high incidence of multidrug-resistant (MDR) organisms, since it is difficult to define the impact of these pathogens on mortality. Although an insight into risk factors for VAP in COVID-19 patients is given in the coVAPid study [6] – a large multicenter retrospective cohort study comparing COVID-19 ICU patients with patients with influenza and no viral infection – a particularly high percentage of MDR pathogens, especially carbapenem-resistant *Acinetobacter baumannii* (CR-Ab) [14], has been documented in countries, such as Italy, already affected by antimicrobial resistance.

Since there is an important knowledge gap regarding the impact and characteristics of MDR-related VAP in pre-COVID-19 and COVID-19 patients, the aim of this monocentric, observational, cohort study is to evaluate the incidence, microbial cause, resistance patters, risk factors and impact on outcome of VAP in a pre-pandemic and a COVID-19 cohort, considering a high MDR incidence context.

## Methods

This mixed prospective and retrospective cohort study was conducted at the “Città della Salute e della Scienza” University Hospital in Turin, Italy, between January 2016 and December 2022. The study was designed and implemented in accordance with the principles stated in the Declaration of Helsinki for biomedical research involving human subjects. The reporting of the study followed the STROBE (STrengthening the Reporting of OBservational studies in Epidemiology) checklist [15]. The study protocol received approval from the Inter-Company Ethics Committee (Study number 0076375 (01/08/16,) and 11,712/2020 (00548/2020). The data collection process was conducted anonymously, and the need for individual consent was waived by the Local Ethics Committee. The consort diagram illustrating the flow of participants is available in the supplementary materials (Supplementary, S1).

### Study design and setting

This single-center, observational, cohort study included two groups:

(1) A pre-pandemic VAP cohort (NON-COVID-19 cohort), a retrospective cohort consisting of adult patients admitted to one of the three intensive care units (ICUs) of the “Città della Salute e della Scienza” University Hospital in Turin (General, Emergency and Cardiosurgical) between June 2016 and March 2018;

(2) A pandemic VAP cohort (COVID-19 cohort), including adult patients admitted to the same hospital and prospectively enrolled between March 2020 and December 2022, with a diagnosis of COVID-19 related pneumonia, confirmed by the Reverse Transcriptase-Polymerase Chain Reaction (RT-PCR) technique on a sample collected from the lower respiratory tract [16], and treated according to internal protocols for managing severe respiratory failure, incorporating the latest guidelines derived from the recent literature on COVID-19 pneumonia [17].

All adult patients diagnosed with ventilator-associated pneumonia (VAP) according to the latest (European Centre for Disease Prevention and Control, ECDC) definitions were included [18] (see below), while patients with an anticipated survival of less than 24 h, pregnant women and patients undergoing mechanical ventilation for ≤ 48 h were excluded.

Patients demographic characteristics, medical history, the duration of mechanical ventilation, and the use of extracorporeal membrane oxygenation (ECMO) or dialysis, if applicable, were collected from medical records. SOFA and SAPS II scores were assessed upon ICU admission.

The primary outcome of the study was to assess the incidence of VAP in the two cohorts of patients.

Patients were followed until hospital discharge to assess the following secondary outcomes: 28-days mortality, 60-days mortality, ICU mortality defined as the all-cause mortality during ICU stay, hospital mortality defined as the all-cause mortality during hospital stay, duration of ICU stay, and duration of hospitalization.

Microbiological samples were collected according to local protocols. Surveillance cultures, such as tracheal aspirate samples, were obtained at defined time intervals (weekly) in the absence of clinical need. Diagnostic cultures were obtained as needed in the presence of a clinical or laboratory suspicion of infection, with the specific type of samples varying according to the clinical context (e.g., bronchoalveolar lavage, bronchial aspirate).

### Definitions

Early- and late-onset VAP were defined using the thresholds recommended by the Infectious Disease Society of America (IDSA)/American Thoracic Society (ATS) and the International ERS/ESICM/ESCMID/ALAT guidelines. Early- and late-onset VAP were defined using a 5 days cut-off [1].

The isolated pathogens were classified as follows^19^: *Multidrug-Resistant (MDR)* if resistant to at least one drug in three or more different antimicrobial categories; *Extensively Drug-Resistant (XDR)* if sensible to only one or two antimicrobial categories, *Pandrug-Resistant (PDR)* if resistant to all agents in all antimicrobial categories. Gram-negative bacteria (GNB) exhibiting resistance to all first-line antimicrobial agents, which for Gram-negative pathogens implies resistance to beta-lactams (including carbapenems) and fluoroquinolones, were defined as *difficult-to-treat (DTR)* [19].

We calculated the crude incidence and the VAP incidence during 1,000 days of mechanical ventilation (MV) in the ICU using the formula [20]:

$$\begin{aligned}&{\text{VAP}}\,\text{i}\text{n}\text{c}\text{i}\text{d}\text{e}\text{n}\text{c}\text{e} \,\text{i}\text{n} \,\text{t}\text{h}\text{e} \,\text{I}\text{C}\text{U}\\&= \left(\frac{\text{N}\text{u}\text{m}\text{b}\text{e}\text{r} \,\text{o}\text{f} \,\text{p}\text{a}\text{t}\text{i}\text{e}\text{n}\text{t}\text{s} \,\text{w}\text{i}\text{t}\text{h} \,\text{V}\text{A}\text{P} \,\text{d}\text{u}\text{r}\text{i}\text{n}\text{g} \,\text{h}\text{o}\text{s}\text{p}\text{i}\text{t}\text{a}\text{l}\text{i}\text{z}\text{a}\text{t}\text{i}\text{o}\text{n}}{\text{M}\text{V} \,\text{d}\text{a}\text{y}\text{s} \,\text{b}\text{e}\text{f}\text{o}\text{r}\text{e} \,\text{V}\text{A}\text{P} \,\text{o}\text{c}\text{c}\text{u}\text{r}\text{r}\text{e}\text{n}\text{c}\text{e}}\right)\\&\times 1000\end{aligned}$$

With the respective 95% CI calculated as follows:

$$\text{IC}= \text{V}\text{A}\text{P}\, \text{i}\text{n}\text{c}\text{i}\text{d}\text{e}\text{n}\text{c}\text{e}\, \text{i}\text{n} \,\text{t}\text{h}\text{e}\, \text{I}\text{C}\text{U}-\left[1.96 \text{x} \left(\text{S}\text{t}\text{a}\text{n}\text{d}\text{a}\text{r}\text{d} \text{e}\text{r}\text{r}\text{o}\text{r}\right)\right]$$

And standard error defined as follows:

$$\begin{aligned}&\text{Standard}\\&= \sqrt{\frac{ \text{VAP\, incidence \,in \,the\, ICU} \times (1000- \text{VAP\,incidence\,in \,the \,ICU)}}{\text{MV\,days \,before\, VAP \,occurrence}}}\end{aligned}$$

Patients receiving drugs such as cyclosporine, azathioprine, tacrolimus, everolimus, mycophenolate mofetil, methotrexate, and steroids before hospital admission were defined as immunosuppressed. Patients with autoimmune diseases such as rheumatoid arthritis, thyroiditis, ulcerative colitis, or those who had undergone solid organ transplantation were also defined as immunodepressed.

### Statistical analysis

The values are expressed as numbers and frequencies or as median and interquartile range, unless otherwise indicated. We performed the Shapiro–Wilk test to assess the distribution of continuous variables. For dichotomous variables, we evaluated the count and their percentage.

To ascertain whether there were statistically significant differences within each risk group—namely COVID versus non-COVID, survivors versus non-survivors, and within sub-analyses such as cohorts of patients undergoing ECMO or RRT—we employed the Chi-square test for dichotomous variables. For continuous variables, we utilized the Wilcoxon test. We considered p-values less than 0.05 as indicative of significance.

We performed a sensitivity analysis to evaluate different risk factors, including the non-VAP population.

We then conducted a survival analysis using the Cox proportional hazards model. We extracted the Hazard Ratio (HR) and the corresponding 95% confidence intervals (CI).

All the analyses and graphs were performed with the open-source RStudio 2022.07.1 [21, 22].

## Results

### General characteristics of the studied populations

Out of 491 patients (*n* = 250 in the NON-COVID group, *n* = 241 in the COVID-19 group), 203 (*n* = 78 in the NON-COVID group, *n* = 125 in the COVID-19 group) were enrolled in the study. The incidence of VAP was 41.3%, with rates of 51.9% and 31.2% in the COVID-19 and NON-COVID-19 populations, respectively (*p* < 0.001, Supplementary Table, S1).

The crude incidence of VAP was 43 per 1000 MV (95% CI 40 to 50), with rates of 66 per 1000 MV (95% CI 55 to 78) in the COVID-19 population and 28 per 1000 MV (95% CI 38 to 50) in the NON-COVID-19 population (Supplementary S2).

Patients with VAP and COVID-19 exhibited a significantly lower median age and BMI compared to those in the NON-COVID-19 group (Table 1). The prevalence of cardiovascular comorbidities and immunosuppression was notably lower in the COVID-19 group compared to the NON-COVID-19 group. Moreover, the utilization of prior immunosuppressive therapies was more common in the NON-COVID-19 group (*p* < 0.001).

**Table 1:** General characteristics of the overall population with ventilator acquired pneumonia

		Study group		
**Characteristic**	**Overall**, *N* = 203^1^	**NON-COVID-19**, *N* = 78^1^	**COVID-19**, *N* = 125^1^	**p-value** ^2^
Sex, male	149 (73%)	55 (71%)	94 (75%)	0.5
Age, years	64 (55, 73)	69 (56, 77)	63 (54, 71)	0.013
BMI, kg/m2	27 (24, 31)	25 (23, 29)	29 (26, 31)	0.003
Solid organ transplant	25 (12%)	19 (24%)	-	
Cardiovascular comorbidities	50 (25%)	29 (37%)	21 (17%)	0.001
Diabetes type II	36 (18%)	10 (13%)	26 (21%)	0.15
CKD	27 (13%)	21 (27%)	6 (4.8%)	< 0.001
Respiratory comorbidities	27 (13%)	13 (17%)	14 (11%)	0.3
Alcohol or drug abusers	7 (3.5%)	2 (2.6%)	5 (4.0%)	0.7
Cirrhosis	3 (1.5%)	3 (3.8%)	0 (0%)	0.055
Immunodepression	16 (7.9%)	10 (13%)	6 (4.8%)	0.039
Immunosuppressive therapy	25 (12%)	19 (24%)	6 (4.8%)	< 0.001
Cyclosporin	8 (3.9%)	8 (10%)	0 (0%)	< 0.001
Tacrolimus/Everolimus	3 (1.5%)	3 (3.8%)	0 (0%)	0.055
Azathioprine	3 (1.5%)	3 (3.8%)	0 (0%)	0.055
Mofetyl Mycophenolate	9 (4.4%)	9 (12%)	0 (0%)	< 0.001
Metothrexate	1 (0.5%)	0 (0%)	1 (0.8%)	> 0.9
Steroids	19 (9.4%)	18 (23%)	1 (0.8%)	< 0.001
ECMO support	56 (28%)	17 (22%)	39 (31%)	0.14
RRT	48 (24%)	32 (41%)	16 (13%)	< 0.001
SAPS II score	52 (42, 58)	54 (43, 62)	51 (41, 57)	0.2
SOFA score	10 (8, 12)	10 (8, 13)	9 (7, 11)	< 0.001

^1^n (%); Median (IQR), ^2^Pearson’s Chi-squared test; Fisher’s exact test, Wilcoxon rank sum test

*Abbreviations* VAP: Ventilator-Associated Pneumonia, COVID-19: Coronavirus Disease 2019, BMI: Body Mass Index, CKD: Chronic Kidney Disease, ECMO: Extracorporeal Membrane Oxygenation, RRT: Renal Replacement Therapy, SAPS II score: Simplified Acute Physiology Score II, SOFA score: Sequential Organ Failure Assessment score

The median SAPS II scores did not show significant differences between the groups (Table 1). However, the median SOFA score upon admission was significantly lower in the COVID-19 group (9, interquartile range: 7–11) compared to the NON-COVID-19 group (10, interquartile range: 8–13) (*p* < 0.001).

In the sensitivity analysis (Supplementary, Table S2), comparing the overall population (*n* = 491) with and without COVID-19, several notable differences were observed. The COVID-19 group had a higher proportion of males (75% vs. 65%, *p* = 0.017) and a lower median age (64 years vs. 69 years, *p* < 0.001). The NON-COVID-19 group had a higher prevalence of cardiovascular comorbidities (38% vs. 19%, *p* < 0.001), while chronic kidney disease (CKD) was more common in the COVID-19 group (7.9% vs. 21%, *p* < 0.001).

*Timing of the first episode of VAP*.

The median time to onset of the first VAP episode was 8 days (interquartile range: 4–11) with no significant differences between COVID-19 and NON-COVID-19 patients (Table 2).

**Table 2:** Characteristics of pathogens in the first episode of ventilator acquired pneumonia

		Study group		
**Characteristic**	**Overall**, *N* = 203^1^	**NON-COVID-19**, *N* = 78^1^	**COVID-19**, *N* = 125^1^	**p-value** ^2^
Time to infection (days)	8 (4, 11)	7 (4, 15)	8 (4, 11)	> 0.9
Number of pathogens determining VAP				< 0.001
0	11 (5%)	10 (13%)	1 (1%)	
1	139 (68%)	45 (58%)	94 (75%)	
2	49 (24%)	21 (27%)	28 (22%)	
3	4 (2%)	2 (3%)	2 (2%)	
Gram-negative related VAP	167 (82%)	67 (86%)	100 (80%)	0.3
Gram-positive related VAP	45 (22%)	7 (9%)	38 (30%)	< 0.001
MDR-related VAP	150 (74%)	59 (76%)	91 (73%)	0.7
XDR-related VAP	94 (46%)	36 (46%)	58 (46%)	> 0.9
PDR-related VAP	3 (1%)	2 (3%)	1 (1%)	0.6
DTR-related VAP	84 (41%)	32 (41%)	52 (42%)	> 0.9
ESBL-related VAP	19 (9%)	13 (17%)	6 (5%)	0.005
Beta-lactams resistance	174 (86%)	67 (86%)	107 (86%)	> 0.9
Carbapenem resistance	106 (52%)	42 (54%)	64 (51%)	0.7
Fluoroquinolones resistance	125 (62%)	52 (67%)	73 (58%)	0.2
Colistin resistance	20 (10%)	13 (17%)	7 (6%)	0.010
CR-Ab	55 (27%)	9 (12%)	46 (37%)	< 0.001
CR-KPC	48 (24%)	27 (35%)	21 (17%)	0.004
MRSA	12 (6%)	2 (3%)	10 (8%)	0.13
*E. coli* ESBL	7 (3.4%)	5 (6.4%)	2 (1.6%)	0.11
*Aspergillus spp.*	3 (1%)	1 (1.5%)	2 (2%)	0.86

^1^n (%); Median (IQR), ^2^Pearson’s Chi-squared test; Fisher’s exact test, Wilcoxon rank sum test

*Abbreviations* MDR: Multi-Drug Resistant, XDR: Extensively Drug Resistant, PDR: Pan Drug Resistant, DTR: Difficult to treat, ESBL: Extended-Spectrum Beta-Lactamase, CR-Ab: *Carbapenem-Resistant Acinetobacter baumannii*, CR-KPC: *Carbapenem-Resistant Klebsiella pneumoniae*, MRSA: Methicillin-Resistant Staphylococcus aureus, E.coli ESBL: Escherichia coli Extended-Spectrum Beta-Lactamase

The number of pathogens causing VAP varied significantly between the groups (*p* < 0.001), with the COVID-19 group having a higher percentage of monomicrobial cases (75%) compared to the NON-COVID-19 group (58%).

When comparing early- and late-onset VAP cases (see Supplementary Table S3), no difference in the number of MDR pathogens could be detected. Looking specifically at XDR and Extended-Spectrum Beta-Lactamase (ESBL) bacteria, higher frequencies in late-onset VAP cases (*p* = 0.043, Supplementary Table S3) were observed. CR-Ab was the only MDR pathogen significantly more common in the late-onset group (*p* = 0.036, Supplementary Table S4).

*Microbiological results*.

The prevalence of Gram-positive bacteria-related VAP was significantly higher in the COVID-19 group (30%) compared to the NON-COVID-19 group (9%) (*p* < 0.001). In contrast, no significant differences in the prevalence of Gram-negative bacteria-related VAP (80% in the COVID-19 group and 86% in the NON-COVID-19 group) were observed. Furthermore, no significant differences concerning the prevalence of VAP related to antibiotic-resistant microorganisms were observed between the two populations. Colistin resistance was higher in the NON-COVID-19 group (17%) compared to the COVID-19 group (6%) (*p* = 0.010).

The most frequently observed MDR pathogens were CR-Ab (overall 27%, COVID-19 37%, NON-COVID-19 12%), CR-KPC (overall 24%, COVID-19 17%, NON-COVID-19 35%), Methicillin-resistant *Staphylococcus aureus* (MRSA) (overall 6%, COVID-19 8%, NON-COVID-19 3%), and ESBL *E.coli* (overall 3.4%, COVID-19 1.6%, NON-COVID-19 6.4%) (Table 2). *Enterobacterales* were isolated in 64% and 34% of VAP episodes in the NON-COVID-19 and COVID-19 population, respectively, ESBL-producing *Enterobacterales* accounting for 14% and 4.8% of all VAP episodes, respectively. *Pseudomonas aeruginosa* was identified in 12% and 14% of VAP episodes in the NON-COVID-19 and COVID-19 group, respectively; MDR *Pseudomonas aeruginosa* accounted for 4% and 3% of all episodes, respectively.

Several specific risk factors for multidrug-resistant high incidence pathogens (i.e. CR-Ab and CR-KPC) were identified in the COVID-19 population with VAP. In the CR-Ab subgroup analysis (Supplementary, table S5), NON-COVID-19 patients had a significantly higher incidence of CKD and higher median SOFA score values compared to COVID-19 patients. In the CR-KPC subgroup analysis (Supplementary table S5), COVID-19 patients showed a higher need for ECMO support compared to NON-COVID-19 patients (48% vs. 11%, *p* = 0.005).

In the difficult-to-treat subgroup analysis [19] (Supplementary, table S6), a significant difference was found between the COVID-19 and NON-COVID-19 subgroups regarding the following characteristics: high BMI (*p* = 0.036), CKD (*p* = 0.018), use of steroids (*p* = 0.004), cyclosporin (*p* = 0.019), and mofetyl mycophenolate (*p* = 0.019), RRT (*p* < 0.001), and high SOFA score (*p* = 0.005).

In the MDR organisms subgroup analysis [23] (Supplementary, table S7), a significant difference was found between COVID-19 and NON-COVID-19 subgroups concerning the following characteristics: high BMI (*p* < 0.001), cardiovascular comorbidities (*p* = 0.018), CKD (*p* < 0.001), immunosuppressive therapy (*p* = 0.001), use of cyclosporine (*p* = 0.001), mofetyl mycophenolate (*p* < 0.001), and steroids (*p* < 0.001), RRT (*p* < 0.001), and high SOFA score (*p* = 0.012).

Other additional subgroup analyses were performed on dialysis and ECMO subgroups (Supplementary, table S9, S10). In the ECMO subgroup, out of a total of 56 patients among the overall 84 (as shown in Table 1 and Supplementary Table S10), 39 were COVID-19 patients, with an incidence of VAP at 67%.

*Survival outcomes*.

As presented in Table 3, no significant difference was observed between the two groups regarding the length of ICU stay in the overall population with VAP. The median length of ICU stay was 26 days for both populations. However, it can benoted that the length of hospital stay was significantly longer for NON-COVID-19 patients, compared to the COVID-19 group (median of 61 days vs. 26 days, respectively). Conversely, the duration of mechanical ventilation did not exhibit a significant difference between the two groups, with a median of 19 days for the overall VAP population.

**Table 3:** Outcomes in the overall population with ventilator acquired pneumonia

		Study group		
**Characteristic**	**Overall**, *N* = 203^1^	**NON-COVID-19**, *N* = 78^1^	**COVID-19**, *N* = 125^1^	**p-value** ^2^
ICU length of stay, days	26 (16, 39)	26 (14, 49)	26 (18, 32)	0.7
Hospital length of stay, days	30 (22, 60)	61 (29, 90)	26 (19, 34)	< 0.001
Duration of MV, days	19 (11, 31)	20 (9, 43)	18 (13, 25)	0.3
Death in ICU	114 (56%)	26 (33%)	88 (71%)	< 0.001
Death in hospital	125 (62%)	33 (43%)	92 (74%)	< 0.001

^1^n (%); Median (IQR), ^2^Pearson’s Chi-squared test; Fisher’s exact test, Wilcoxon rank sum test

Mortality rates were higher in the COVID-19 group, accounting for 71% in the ICU, 74% in the hospital, and 72% at 60 days (Table 3).

When comparing early- versus late-onset VAP, no differences in patient outcomes were observed; however, variations were noted concerning ICU length of stay and duration of mechanical ventilation (Supplementary Table S3).

When evaluating the overall population (combining VAP and non-VAP cases), it is observed that VAP is associated with increased mortality in both the ICU and hospital settings, as detailed in Table S1.

*Cox proportional hazard model and survival analysis*.

**Fig. 1:**
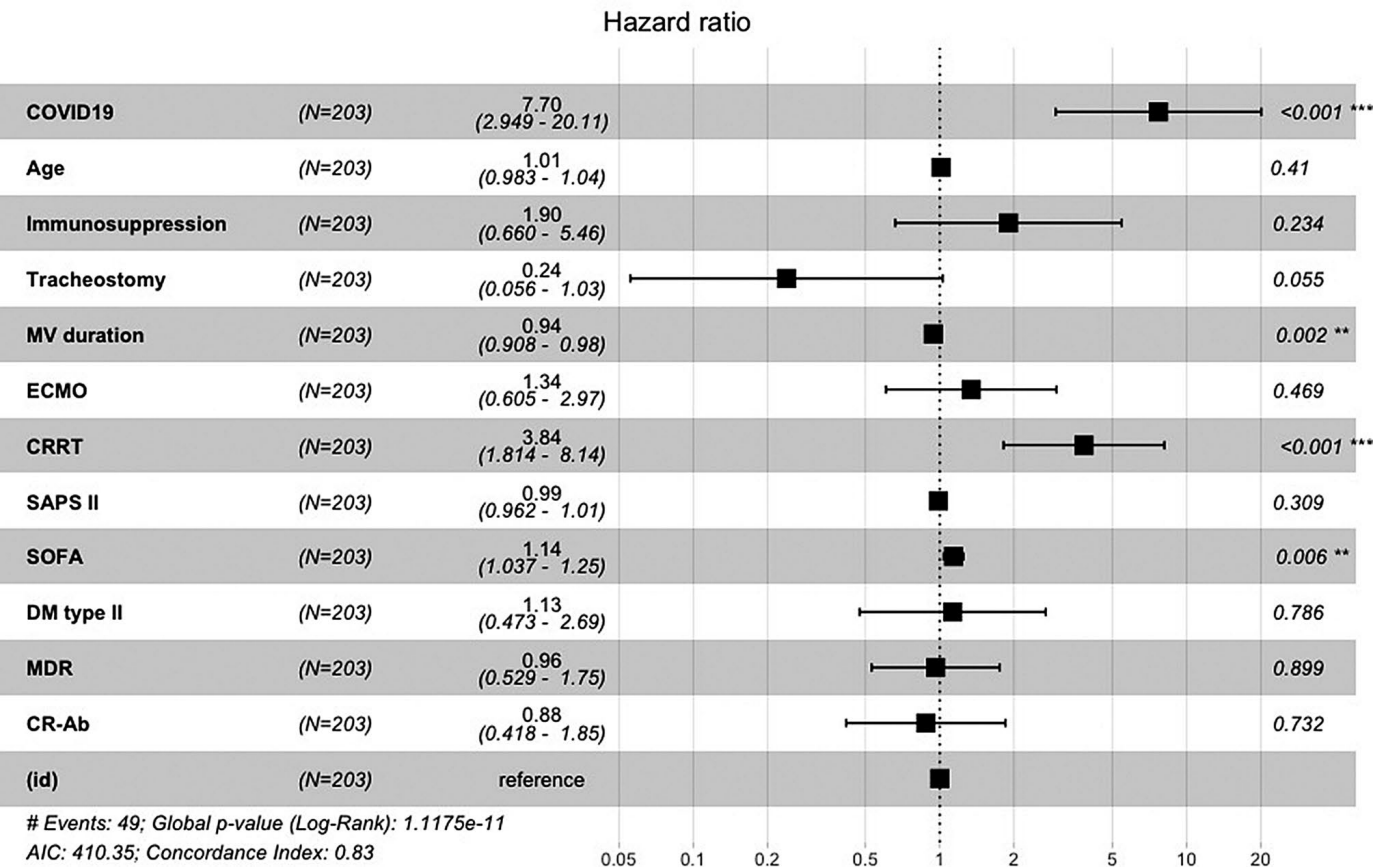
Forest Plot representing the Cox proportional hazards survival analysis for the population with Ventilator-Associated Pneumonia (VAP). The dashed line represents a Hazard Ratio of 1

Among the variables identified from the analysis comparing survivors and non-survivors (Supplementary, S8), the presence of COVID-19 was significantly associated with an increased risk of 28-day ICU mortality, demonstrating a hazard ratio (HR) of 7.95 (95% CI 3.10-20.36, *p* < 0.001) (Fig. 1).

Tracheostomy (HR = 0.22, 95% CI 0.05–0.93, *p* = 0.04) and a shorter duration of mechanical ventilation (HR = 0.94, 95% CI 0.90–0.97, *p* < 0.001) were associated with a reduced hazard of 28-day all-cause mortality. High SOFA score values at admission (HR = 1.11, 95% CI 1.01–1.22, *p* = 0.024) and the need for dialysis (HR = 3.34, 95% CI 1.59–7.02, *p* < 0.001) were significantly associated with an increased hazard of 28-day mortality. However, other variables such as age, immunosuppressive therapy, SAPS II score, ECMO support, and comorbidities (i.e. diabetes mellitus), or specific pathogens such as MDR and CR-Ab, did not exhibit a significant association with the outcome.

**Fig. 2:**
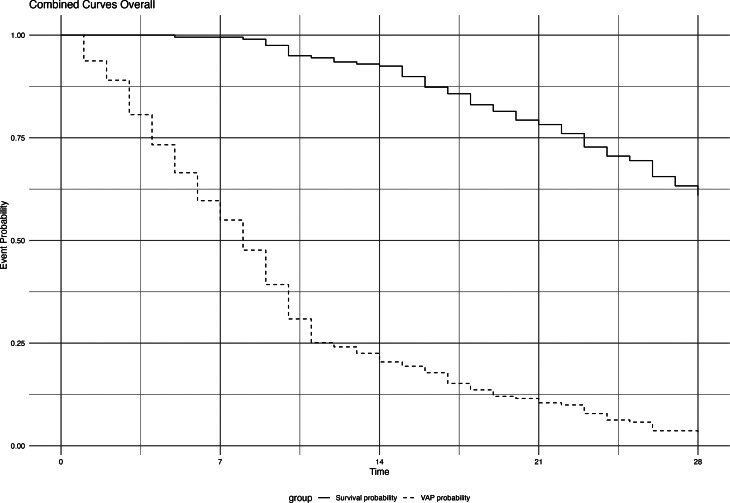
Survival Curves of the Event (Death or Infection). The graph displays the incidence of 28-day ICU survival (solid red line) and the probability of experiencing a Ventilator-Associated Pneumonia (VAP) (dashed blue line) within 28 days in the overall population, the population with COVID-19, and the population without COVID-19

Considering the timing of VAP, this infection occurred around the eighth day. This model appears to hold true for both patients with and without COVID-19 (Fig. 2).

**Discussion**.

The results of the present study suggest that: (1) VAP is one of the major complications among ventilated patients, with values comparable to those already known in the literature, particularly for the COVID-19 cohort; (2) the incidence of VAP is higher in COVID-19 patients; (3) COVID-19 is associated with a significantly increased risk of 28-day mortality in patients with VAP, regardless of whether the pathogen is MDR or DTR; (4) tracheostomy and a shorter duration of mechanical ventilation have a protective effect against mortality during VAP, while dialysis and a high SOFA score at admission seem to worsen the outcome; (5) the incidence of multidrug-resistant pathogens is high in both pandemic and pre-pandemic periods without a significant difference overall.

Considering microbiology, and specifically MDR organisms, CR-KPC was more frequent in the pre-pandemic period while CR-Ab was more frequent in the pandemic period, but either factor has no direct impact on mortality.

Overall, the incidence of VAP is in line with what has been described in the literature, ranging from 48–79% [3, 4, 6, 24–26]. The overall incidence of VAP per 1,000 MV days is similar to that reported in the literature.

Data relating to SARS-CoV-2 patients highlight results that differ significantly from those reported in a multicenter study conducted during the first wave of COVID-19 in Italy, which reported a rate of 29% [10]. On the other hand, a European multicenter study confirmed our findings, reporting an incidence of 42–89% [6]. Similarly, the incidence of VAP/1000 MV days in the NON-COVID-19 and COVID-19 population, although high, is in line with what has been described in the literature [5, 20, 24, 27, 28].

In our population COVID-19 disease appeared to have a greater impact (HR 7.95 95% CI 3.10-20.36) compared to what has been reported in the previous literature [3, 9], probably due to the particularly high patients severity.

It has also been highlighted that immunosuppression does not appear to be associated with 28-day mortality, in contrast to the findings of the previous study by Maes et al. [3], reporting a higher prevalence of infections among COVID-19 patients, and supporting the hypothesis of a significant burden of immunoparesis in this group. This might be explained by the high number of patients in the NON-COVID-19 cohort already undergoing immunosuppressive therapy due to solid organ transplant (24% of the non-COVID-19 patients, Table 1).

Considering the timing of VAP, this infection occurred around the eighth day. This model appears to hold true for both populations, with and without COVID-19 (Fig. 2), and confirms literature data. Obviously, the number of days of mechanical ventilation resulted inversely proportional to the risk of death at 28 days.

The results of our study’s did not confirm a high prevalence of MDR pathogens and MRSA in late-onset cases (Supplementary, table S3) [28, 29], but only of XDR microorganisms, such as CR-Ab [28, 29].

In contrast to what has been reported in the literature, we did not find a relationship with mortality [28, 29], possibly due to the small sample size or the high complexity of selected patients (Supplementary, table S3). Nonetheless, we identified variations in ICU stay and mechanical ventilation duration (Supplementary, table S3).

The timing of tracheostomy in COVID-19 settings has been discussed in the literature, but data are inconclusive [30]. Our data seems to confirm that tracheostomy appears to reduce the incidence of VAP [31].

SOFA score at admission emerged as a significant parameter in the Cox proportional hazard model test; however, its impact on the 28-day mortality risk is weak (HR 1.11), probably due to the lack of time-varying values. While the usefulness of the SOFA score appears to be a matter of debate in some cases [32], there are studies suggesting its usefulness at the time of admission [12], or when monitored over time [13], as a predictive indicator of mortality or VAP.

Dialysis and renal pathology as predisposing factors for the development of ventilator-associated events have already been addressed by the literature but only in the pediatric ICU population [33]. Our findings seem to confirm how this association is linked to a condition of fluid overload which can predispose to pulmonary complications. Additionally, previous studies have shown that renal pathology, specifically an increase in estimated glomerular filtration rate, is associated with a higher risk of MDR bacterial infection [34]. In our study, we observed that 36 out of the patients undergoing dialysis (75%) had MDR infections (Supplementary, S9), indicating a relatively high percentage, but not significantly higher compared to the non-dialysis group (*n* = 114, 74%) (Supplementary, S9).

As far as the ECMO subgroup in concerned, the incidence of VAP was 67%, in line with the available literature [35]. Our findings revealed a notable incidence of late-onset VAP among ECMO patients (*p* = 0.011, Supplementary, table S3), implying a predisposition for delayed pulmonary infection in this subgroup. This phenomenon could potentially be attributed to the ultra-protective ventilation strategy employed for ECMO patients [36, 37], which potentially minimizes lung inflammation [38] and reduces the chances of developing acute infections.

While the univariate analysis suggests that ECMO had a significant impact on survival in VAP patients (*p* = 0.12), the Cox model failed to confirm these findings (Fig. 1). The ECMO subgroup showed a high mortality rate (66% vs. 56% in the overall population), possibly due to the severity of patients and specific factors dependent on extracorporeal support (altered distribution of microbiological agents for molecular size, protein binding or lipophilicity [39], age of the circuit [40], type of membrane, hemodiluiton [41]). Interestingly, we found no susceptibility to MDR pathogens or other bacteria in this population (Supplementary, S10).

*Impact of SARS-CoV-2 infection on VAP micro-organisms*.

Gram-negative bacteria represented the majority of isolates in our population (86% in the NON-COVID-19 cohort and 80% in the COVID-19 cohort, Table 2), which is consistent with the available literature [26].

As for Gram-positive bacteria, they were significantly more prevalent among COVID-19 patients, in line with literature findings describing a higher frequency of Gram-positive bacterial secondary infections in patients with infections of viral origin. The most frequent Gram-positive bacteria in the overall population was Methicillin-resistant *Staphylococcus aureus* [overall, *N* = 12 (6%), COVID-19 *n* = 10 (8%), NON-COVID-19 *n* = 2 (3%)]. Its high incidence in the COVID-19 population well supports the findings previously observed after viral infections, as demonstrated before with influenza and MERS, and reaffirmed with SARS-CoV-2 [42–44].

We compared our results with those of the coVAPid study [9], which is a large multicenter retrospective study comparing a cohort of COVID-19 ICU patients with a cohort with influenza and another one with neither of these conditions. Significant differences emerged, especially in terms of local ecology. Specifically, the MRSA incidence rate in the COVID-19 population of the coVAPid study was 2.9% [9], while ours reached 8%. When evaluating the impact of MDR pathogens exclusively in the COVID-19 population, the coVAPid study showed an incidence of 20.7% [9], whereas ours was 73%. Despite these high rates of MDR, DTR bacteria, and antibiotic resistance, we found no differences in survival, suggesting that COVID-19 infection itself might represents a fundamental determinant of mortality, regardless of microbiological isolates and its antimicrobial susceptibility.

**Fig. 3:**
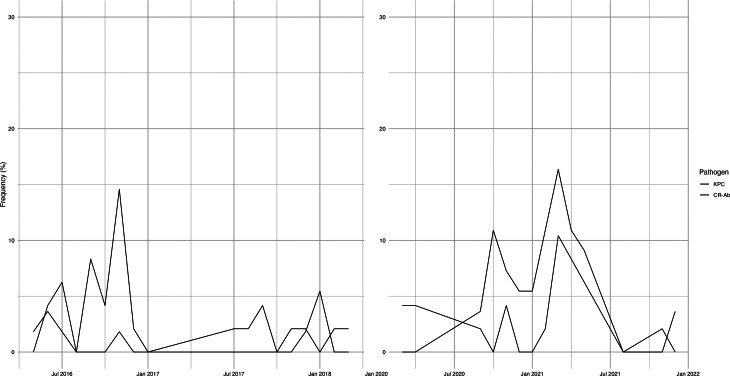
Distribution of frequencies over time of *Carbapenem-Resistant Klebsiella pneumoniae* (CR-KPC) and *Carbapenem-Resistant Acinetobacter baumannii* (CR-Ab). The frequencies were calculated by evaluating the incidence of each individual pathogen within a specific timeframe (a month) relative to the overall period and the total number of ventilator-acquired pneumonia episodes caused by that specific pathogen

With regard more specifically to local microbiology, we found a higher incidence of MDR pathogens compared to the literature, given that the 150 first episodes of VAP (61%) were determined by MDR bacteria [*n* = 59 (75.6%), NON-COVID-19: *n* = 91 (72.8%), COVID-19] [10]. In particular, CR-KPC (overall *n* = 27, 35%, Table 2) was the more frequent MDR isolate in the NON-COVID-19 population peaking at 14.6% of the overall CR-KPC-related VAP episodes during November 2016 (Fig. 3).

Interestingly, this MDR-pathogen was the first to be isolated in our center in the COVID-19 cohort as well [45]. Several factors may explain this phenomenon: frequent transfers of patients between hospitals and wards; low initial availability of personal protective equipment; extreme COVID-19 patients severity prior to vaccinations; use of immunomodulatory and immunosuppressant therapies; wide and liberal use of early empirical antibiotic therapy. Furthermore, in the COVID-19 population, we observed a higher incidence of MDR *Pseudomonas aeruginosa* (3%) but a lower incidence of ESBL-producing *Enterobacterales* (4.8%) compared to the literature (0.4% and 7.4%, respectively) [24].

Regarding the COVID-19 population, we found a high incidence of CR-Ab (37%, Table 2) peaking at 16.4% of the overall CR-Ab-related VAP episodes between February and March 2021 (Fig. 3) [45]. This pathogen has been widely described in Italy among COVID-19 patients, and in particular in Piedmont, where it has probably caused a real cluster, with important critical issues in terms of effective therapies and infection control measures [14]. Neither CR-Kp KPC nor CR-Ab were found to correlate with 28-day all-cause mortality. The high incidence of these pathogens must represent a constant alert on the need to consider the local microbiological ecology and strictly apply all infection control measures. The lack of a direct impact on mortality would seem to at least partially reassure about the promptness and appropriateness of clinical management.

Among the limitations of the present study, first of all, it should be noted that it is a single-center experience, which may limit the generalizability of the results to other hospital settings. Secondly, the COVID-19 and NON-COVID-19 populations significantly differ in terms of age and BMI. However, we conducted sensitivity analyses to address these differences and ensure robust results (Supplementary, table S2). Thirdly, the NON-COVID-19 cohort originates from different intensive care contexts, including cardiac-ICU patients with their specific characteristics. Nevertheless, the presence of very severe patients and immunosuppressed heart and lung transplanted patients facilitated the comparison between the populations, given their severe immunosuppression and the compromised lung function. Another limitation is the temporal gap between the enrollment of the two cohorts, which introduced some heterogeneity in the clinical management, such as the use of different antibiotic therapies. The high incidence of MDR organisms in the pre-pandemic phase made it challenging to distinguish the local ecological impact from specific risk factors in the study population. As a result, we performed ad hoc analyses for individual pathogens to address this challenge (Supplementary, tables S4, S5, S6, S7). Another limitation is that the non-COVID19 cohort consists of retrospectively enrolled patients, which may introduce biases.

## Conclusions

The present study offers valuable insights into the impact of COVID-19 on VAP mortality, regardless of the presence of MDR or DTR pathogens. This highlights the importance of carefully managing VAP in COVID-19 patients, even in the absence of MDR organisms, albeit in a high prevalence setting. Furthermore, our findings seem to confirm a high incidence of Gram-positive bacteria in the post-viral population.

As far as MDR pathogens are concerned, although specific pathogens – such as CR-KPC and CR-Ab – showed variations between the two time periods, they did not directly influence the 28-day all-cause mortality. However, the study reiterates the need to understand the local ecology to promptly and adequately manage these infections and limit the spread of MDR cases with adequate infection control measures.

Among the factors significantly affecting VAP outcomes, tracheostomy and a shorter mechanical ventilation durations were associated with a protective effect against VAP mortality, while high SOFA scores and dialysis appear to negatively impact on patients outcomes.

### Electronic supplementary material

Below is the link to the electronic supplementary material.

Supplementary Material 1

## Data Availability

The dataset is available from the corresponding author on reasonable request.

## Declarations

The study protocol received approval from the Inter-Company Ethics Committee (Study Number 00548/2020). The data collection process was conducted anonymously, and the need for individual consent was waived by the Local Ethics Committee.
