# Supplementary material for: Multidrug-resistant pathogens and ventilator-associated pneumonia in critically ill COVID-19 and non-COVID-19 patients: a prospective observational monocentric comparative study
Source: Respir Res. 2024 Apr 18;25:168. doi: 10.1186/s12931-024-02779-1 (PMC11027225; doi:10.1186/s12931-024-02779-1)
Supplement: Supplementary file 1 — Supplementary Material 1 [file 12931_2024_2779_MOESM1_ESM.docx]

**Multidrug-Resistant Pathogens and Ventilator-Associated Pneumonia in Critically Ill COVID-19 and Non-COVID-19 Patients: A Mixed Prospective and Retrospective Cohort Monocentric Study**

Supplementary materials

Summary

S1. CONSORT flow diagram.

S2. Calculations of the VAP/1000 MV days.

Tables

S1. Analysis between VAP and non-VAP population. General characteristics of the overall population and survival outcomes.

S2. Sensitivity analysis. General characteristics of the overall population.

S3. Early versus late ventilator-associated pneumonia.

S4. General characteristics of the population positive to CR-Ab during the first episode of VAP.

S5. General characteristics of the population positive to CR-KPC during the first episode of VAP.

S6. General characteristics of the population positive to difficult to treat (DTR) gram-negative bacteria during the first episode of VAP.

S7. General characteristics of the population positive to multi-drug resistant organisms (MDROs) during the first episode of VAP.

S8. General characteristics among survivors and non survivors in VAP population.

S9. General characteristics of pathogens in the first episode of ventilator acquired pneumonia among dialysis and non-dialysis patients.

S10. General characteristics of pathogens in the first episode of ventilator acquired pneumonia among ECMO and non ECMO patients.

S1. CONSORT flow diagram.


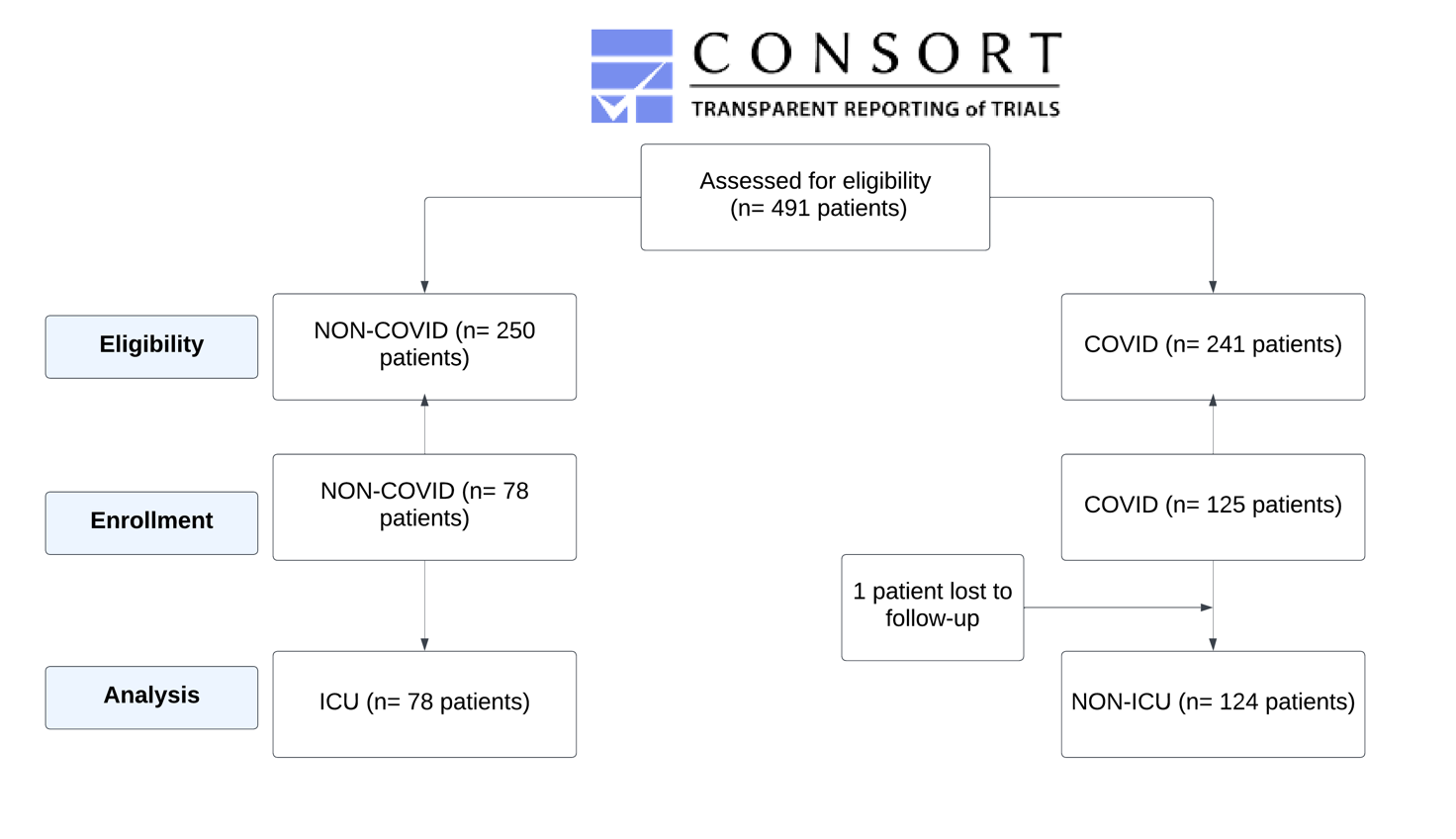


S2. Calculations of the VAP/1000 MV days.

**Overall population**

$$Number of patients with VAP during hospitalization= 203$$

$$MV days before VAP occurrence= 4634$$

$$VAP incidence in the ICU= \left( \frac{203}{4634} \right)x 1000=43.80$$

$$Standard error= \sqrt{\frac{VAP incidence in the ICU x \left( 1000- VAP incidence in the ICU \right)}{MV days before VAP occurrence}}$$

$Standard error= \sqrt{\frac{43.80 x \left( 1000- 43.80 \right)}{4634}}$=$\sqrt{9.04}=3.01$

IC_low_ = $43.80-\left[ 1.96 x \left( 3.01 \right) \right]=$37.9

IC_high_ = $43.80+\left[ 1.96 x \left( 3.01 \right) \right]=$49.7

**NON COVID population**

$$Number of patients with VAP during hospitalization= 78$$

$$MV days before VAP occurrence= 2752$$

$$VAP incidence in the ICU= \left( \frac{78}{2752} \right)x 1000=28.34$$

$Standard error= \sqrt{\frac{28.34 x \left( 1000- 28.34 \right)}{2752}}$=$\sqrt{10.1}=$3.18

IC_low_ = $28.34-\left[ 1.96 x \left( 3.18 \right) \right]=$37.9

IC_high_ = $28.34+\left[ 1.96 x \left( 3.18 \right) \right]=$49.7

**COVID population**

$$Number of patients with VAP during hospitalization= 125$$

$MV days before VAP occurrence=$1882

$$VAP incidence in the ICU= \left( \frac{125}{1882} \right)x 1000=66.42$$

$Standard error= \sqrt{\frac{66.42 x \left( 1000- 66.42 \right)}{1882}}$=$\sqrt{32.95}=5.74$

IC_low_ = $66.42-\left[ 1.96 x \left( 5.74 \right) \right]=55.17$

IC_high_ = $66.42+\left[ 1.96 x \left( 5.74 \right) \right]=$77.67

S1. Analysis between VAP and non-VAP population. General characteristics of the overall population and survival outcomes.

| **Characteristic** | **Overall**, N = 491 | **VAP group** | | **p-value***^1^* |
| --- | --- | --- | --- | --- |
|  |  | **No**, N = 288 | **Yes**, N = 203 |  |
| COVID-19, n (%) | 241 (49) | 116 (40) | 125 (62) | <0.001 |
| Sex, male, n (%) | 342 (70) | 193 (67) | 149 (73) | 0.13 |
| Age, years, Median (IQR) | 67 (57 – 75) | 69 (58 – 77) | 64 (55 – 73) | 0.003 |
| BMI, kg/m2, Median (IQR) | 27 (24 – 30) | 27 (24 – 30) | 27 (24 – 31) | 0.49 |
| Diabetes type II, n (%) | 105 (21) | 69 (24) | 36 (18) | 0.10 |
| CKD, n (%) | 71 (14) | 44 (15) | 27 (13) | 0.54 |
| Respiratory comorbidities, n (%) | 79 (16) | 52 (18) | 27 (13) | 0.16 |
| Alcohol or drug abusers, n (%) | 23 (4.7) | 16 (5.6) | 7 (3.5) | 0.28 |
| Immunodepression, n (%) | 43 (8.8) | 27 (9.4) | 16 (7.9) | 0.56 |
| Immunosuppressive therapy, n (%) | 65 (13) | 40 (14) | 25 (12) | 0.61 |
| ECMO support, n (%) | 84 (17) | 28 (9.8) | 56 (28) | <0.001 |
| RRT, n (%) | 90 (18) | 42 (15) | 48 (24) | 0.011 |
| Steroids, n (%) | 44 (9.0) | 25 (8.7) | 19 (9.4) | 0.80 |
| Concomitant BSI/CRBSI, n (%) | 95 (26) | 35 (22) | 60 (30) | 0.12 |
| SAPS II score, Median (IQR) | 52 (40 – 61) | 52 (40 – 63) | 52 (42 – 58) | 0.69 |
| SOFA score, Median (IQR) | 9 (7 – 12) | 9 (6 – 12) | 10 (8 – 12) | 0.24 |
| ICU length of stay, days, Median (IQR) | 16 (9 – 28) | 11 (7 – 19) | 26 (16 – 39) | <0.001 |
| Hospital length of stay, days, Median (IQR) | 29 (17 – 51) | 27 (15 – 48) | 30 (22 – 60) | 0.002 |
| Duration of MV, days, Median (IQR) | 10 (5 – 21) | 7 (4 – 12) | 19 (11 – 31) | <0.001 |
| ICU mortality, n (%) | 208 (43) | 94 (33) | 114 (56) | <0.001 |
| Hospital mortality, n (%) | 239 (49) | 114 (40) | 125 (62) | <0.001 |
| Death at Day 60, n (%) | 217 (44) | 106 (37) | 111 (55) | <0.001 |
| *^1^* Wilcoxon rank sum test; Pearson’s Chi-squared test | | | | |

S2. Sensitivity analysis. General characteristics of the overall population.

|  | | **Study group** | |  | |
| --- | --- | --- | --- | --- | --- |
| **Characteristics** | **Overall**, N = 491^1^ | **NON-COVID-19,** N= 250^1^ | **COVID-19,** N = 241^1^ | **p-value**^2^ | |
| Sex, male | 342 (70%) | 162 (65%) | 180 (75%) | 0.017 | |
| Age, years | 67 (57, 75) | 69 (58, 79) | 64 (56, 72) | <0.001 | |
| BMI, kg/m2 | 27 (24, 30) | 26 (23, 29) | 28 (25, 31) | <0.001 | |
| Cardiovascular comorbidities | 141 (29%) | 95 (38%) | 46 (19%) | <0.001 | |
| Diabetes type II | 105 (21%) | 49 (20%) | 56 (23%) | 0.3 | |
| CKD | 71 (14%) | 52 (21%) | 19 (7.9%) | <0.001 | |
| Respiratory comorbidities | 79 (16%) | 47 (19%) | 32 (13%) | 0.10 | |
| Alcohol or drug abusers | 23 (4.7%) | 15 (6.0%) | 8 (3.3%) | 0.2 | |
| Cirrhosis | 20 (4.1%) | 16 (6.4%) | 4 (1.7%) | 0.008 | |
| Immunodepression | 43 (8.8%) | 25 (10%) | 18 (7.5%) | 0.3 | |
| Immunosuppressive therapy | 65 (13%) | 47 (19%) | 18 (7.5%) | <0.001 | |
| Cyclosporin | 18 (3.7%) | 18 (7.2%) | 0 (0%) | <0.001 | |
| Tacrolimus/Everolimus | 14 (2.9%) | 11 (4.4%) | 3 (1.2%) | 0.036 | |
| Azathioprine | 4 (0.8%) | 4 (1.6%) | 0 (0%) | 0.12 | |
| Mofetyl Mycophenolate | 28 (5.7%) | 25 (10%) | 3 (1.2%) | <0.001 | |
| Metothrexate | 3 (0.6%) | 2 (0.8%) | 1 (0.4%) | >0.9 | |
| Steroids | 44 (9.0%) | 38 (15%) | 6 (2.5%) | <0.001 | |
| ECMO support | 84 (17%) | 32 (13%) | 52 (22%) | 0.009 | |
| RRT | 90 (18%) | 62 (25%) | 28 (12%) | <0.001 | |
| SAPS II score | 52 (40, 61) | 54 (40, 63) | 48 (40, 58) | 0.2 | |
| SOFA score | 9 (7, 12) | 10 (8, 12) | 9 (7, 11) | 0.006 | |
| ^1^n (%); Median (IQR), ^2^Pearson's Chi-squared test; Fisher's exact test, Wilcoxon rank sum test. | | | | |  |

Abbreviations: VAP: Ventilator-Associated Pneumonia, COVID-19: Coronavirus Disease 2019, BMI: Body Mass Index, CKD: Chronic Kidney Disease, ECMO: Extracorporeal Membrane Oxygenation, RRT: Renal Replacement Therapy, SAPS II score: Simplified Acute Physiology Score II, SOFA score: Sequential Organ Failure Assessment score.

S4. Early versus late ventilator-associated pneumonia.

|  | | **Timing group** | |  |
| --- | --- | --- | --- | --- |
| **Characteristics** | **Overall**, N = 193^1^ | **Early**, N = 53^1^ | **Late**, N = 140^1^ | **p-value**^2^ |
| COVID-19 | 119 (62%) | 27 (51%) | 92 (66%) | 0.060 |
| Sex, male | 143 (74%) | 40 (75%) | 103 (74%) | 0.8 |
| Age, years | 64 (55, 73) | 66 (55, 76) | 64 (55, 72) | 0.2 |
| BMI, kg/m2 | 27 (24, 31) | 26 (23, 30) | 27 (25, 31) | 0.2 |
| Diabetes type II | 32 (17%) | 8 (15%) | 24 (17%) | 0.7 |
| CKD | 26 (13%) | 11 (21%) | 15 (11%) | 0.068 |
| Respiratory comorbidities | 24 (12%) | 10 (19%) | 14 (10%) | 0.10 |
| Alcohol or drug abusers | 7 (3.6%) | 2 (3.8%) | 5 (3.6%) | >0.9 |
| Immunodepression | 14 (7.3%) | 2 (3.8%) | 12 (8.6%) | 0.4 |
| Immunosuppressive therapy | 23 (12%) | 7 (13%) | 16 (11%) | 0.7 |
| ECMO support | 55 (28%) | 8 (15%) | 47 (34%) | 0.011 |
| RRT | 44 (23%) | 15 (28%) | 29 (21%) | 0.3 |
| Steroids | 17 (8.8%) | 6 (11%) | 11 (7.9%) | 0.6 |
| Concomitant BSI/CRBSI | 55 (28%) | 11 (21%) | 44 (31%) | 0.14 |
| SAPS II score | 52 (41, 58) | 51 (35, 59) | 52 (43, 58) | 0.3 |
| SOFA score | 10 (8, 12) | 10 (9, 13) | 9 (8, 12) | 0.032 |
| Number of pathogens determining VAP |  |  |  | 0.4 |
| 0 | 7 (4%) | 2 (4%) | 5 (4%) |  |
| 1 | 135 (70%) | 33 (62%) | 102 (73%) |  |
| 2 | 47 (24%) | 16 (30%) | 31 (22%) |  |
| 3 | 4 (2%) | 2 (4%) | 2 (1%) |  |
| Gram-negative related VAP | 164 (85%) | 46 (87%) | 118 (84%) | 0.7 |
| Gram-positive related VAP | 41 (21%) | 14 (26%) | 27 (19%) | 0.3 |
| MDR-related VAP | 147 (76%) | 40 (75%) | 107 (76%) | 0.9 |
| XDR-related VAP | 92 (48%) | 19 (36%) | 73 (52%) | 0.043 |
| PDR-related VAP | 3 (2%) | 0 (0%) | 3 (2%) | 0.6 |
| DTR-related VAP | 82 (42%) | 18 (34%) | 64 (46%) | 0.14 |
| ESBL-related VAP | 19 (10%) | 9 (17%) | 10 (7%) | 0.041 |
| Cephalosporins resistance | 120 (62%) | 31 (58%) | 89 (64%) | 0.5 |
| Betalactams resistance | 170 (88%) | 45 (85%) | 125 (89%) | 0.4 |
| Carbapenem resistance | 104 (54%) | 23 (43%) | 81 (58%) | 0.072 |
| Fluoroquinolones resistance | 122 (63%) | 28 (53%) | 94 (67%) | 0.066 |
| Colistin resistance | 20 (10%) | 5 (9%) | 15 (11%) | 0.8 |
| CR-Ab | 54 (28%) | 9 (17%) | 45 (32%) | 0.036 |
| KPC | 46 (24%) | 10 (19%) | 36 (26%) | 0.3 |
| MRSA | 11 (6%) | 4 (8%) | 7 (5%) | 0.5 |
| ICU length of stay, days | 26 (17, 39) | 18 (13, 27) | 28 (19, 44) | <0.001 |
| Hospital length of stay, days | 30 (22, 60) | 27 (16, 52) | 32 (24, 61) | 0.070 |
| Duration of MV, days | 19 (12, 31) | 15 (10, 25) | 20 (13, 36) | 0.006 |
| ICU mortality | 107 (56%) | 28 (54%) | 79 (56%) | 0.7 |
| Hospital mortality | 118 (62%) | 32 (63%) | 86 (61%) | 0.9 |
| ^1^n (%); Median (IQR) | | | | |
| ^2^Pearson's Chi-squared test; Wilcoxon rank sum test; Fisher's exact test | | | | |

Abbreviations: VAP: Ventilator-Associated Pneumonia, COVID-19: Coronavirus Disease 2019, BMI: Body Mass Index, CKD: Chronic Kidney Disease, ECMO: Extracorporeal Membrane Oxygenation, RRT: Renal Replacement Therapy, SAPS II score: Simplified Acute Physiology Score II, SOFA score: Sequential Organ Failure Assessment score.

S5. General characteristics of the population positive to CR-Ab during the first episode of VAP.

|  | | **Study group** | |  |  |
| --- | --- | --- | --- | --- | --- |
| **Characteristics** | **Overall**, N = 55^1^ | **NON-COVID-19**, N = 9^1^ | **COVID-19**, N = 46^1^ | **p-value**^2^ |  |
| Sex, male | 42 (76%) | 6 (67%) | 36 (78%) | 0.4 |  |
| Age, years | 64 (57, 72) | 52 (43, 64) | 64 (60, 72) | 0.093 |  |
| BMI, kg/m2 | 27 (24, 31) | 23 (19, 24) | 28 (25, 31) | 0.6 |  |
| Cardiovascular comorbidities | 15 (27%) | 4 (44%) | 11 (24%) | 0.2 |  |
| Diabetes type II | 12 (22%) | 0 (0%) | 12 (26%) | 0.2 |  |
| CKD | 5 (9.1%) | 3 (33%) | 2 (4.3%) | 0.027 |  |
| Respiratory comorbidities | 6 (11%) | 0 (0%) | 6 (13%) | 0.6 |  |
| Alcohol or drug abusers | 1 (1.9%) | 0 (0%) | 1 (2.2%) | >0.9 |  |
| Cirrhosis | 0 (0%) | 0 (0%) | 0 (0%) |  |  |
| Immunodepression | 5 (9.1%) | 1 (11%) | 4 (8.7%) | >0.9 |  |
| Immunosuppressive therapy | 5 (9.1%) | 1 (11%) | 4 (8.7%) | >0.9 |  |
| Cyclosporin | 0 (0%) | 0 (0%) | 0 (0%) |  |  |
| Tacrolimus/Everolimus | 0 (0%) | 0 (0%) | 0 (0%) |  |  |
| Azathioprine | 0 (0%) | 0 (0%) | 0 (0%) |  |  |
| Mofetyl Mycophenolate | 0 (0%) | 0 (0%) | 0 (0%) |  |  |
| Metothrexate | 1 (1.8%) | 0 (0%) | 1 (2.2%) | >0.9 |  |
| Steroids | 2 (3.6%) | 1 (11%) | 1 (2.2%) | 0.3 |  |
| ECMO support | 16 (29%) | 3 (33%) | 13 (28%) | 0.7 |  |
| RRT | 7 (13%) | 4 (44%) | 3 (6.5%) | 0.010 |  |
| SAPS II score | 48 (35, 54) | 48 (41, 50) | 48 (35, 54) | 0.8 |  |
| SOFA score | 10 (8, 12) | 12 (11, 13) | 10 (7, 11) | 0.023 |  |
| ^1^n (%); Median (IQR), ^2^Pearson's Chi-squared test; Fisher's exact test, Wilcoxon rank sum test. | | | | | |

Abbreviations: VAP: Ventilator-Associated Pneumonia, COVID-19: Coronavirus Disease 2019, BMI: Body Mass Index, CKD: Chronic Kidney Disease, ECMO: Extracorporeal Membrane Oxygenation, RRT: Renal Replacement Therapy, SAPS II score: Simplified Acute Physiology Score II, SOFA score: Sequential Organ Failure Assessment score.

S6. General characteristics of the population positive to CR-KPC during the first episode of VAP.

|  | | **Study group** | |  |  |
| --- | --- | --- | --- | --- | --- |
| **Characteristic** | **Overall**, N = 48^1^ | **NON-COVID-19**, N = 27^1^ | **COVID-19**, N = 21^1^ | **p-value**^2^ |  |
| Sex, male | 32 (67%) | 19 (70%) | 13 (62%) | 0.5 |  |
| Age, years | 64 (57, 71) | 66 (56, 74) | 64 (57, 70) | 0.9 |  |
| BMI, kg/m2 | 27 (25, 31) | 26 (24, 30) | 29 (26, 35) | 0.8 |  |
| Cardiovascular comorbidities | 13 (27%) | 8 (30%) | 5 (24%) | 0.7 |  |
| Diabetes type II | 9 (19%) | 4 (15%) | 5 (24%) | 0.5 |  |
| CKD | 6 (13%) | 6 (22%) | 0 (0%) | 0.029 |  |
| Respiratory comorbidities | 6 (13%) | 6 (22%) | 0 (0%) | 0.029 |  |
| Alcohol or drug abusers | 1 (2.1%) | 1 (3.7%) | 0 (0%) | >0.9 |  |
| Cirrhosis | 1 (2.1%) | 1 (3.7%) | 0 (0%) | >0.9 |  |
| Immunodepression | 3 (6.3%) | 3 (11%) | 0 (0%) | 0.2 |  |
| Immunosuppressive therapy | 7 (15%) | 7 (26%) | 0 (0%) | 0.014 |  |
| Cyclosporin | 5 (10%) | 5 (19%) | 0 (0%) | 0.059 |  |
| Tacrolimus/Everolimus | 0 (0%) | 0 (0%) | 0 (0%) |  |  |
| Azathioprine | 1 (2.1%) | 1 (3.7%) | 0 (0%) | >0.9 |  |
| Mofetyl Mycophenolate | 5 (10%) | 5 (19%) | 0 (0%) | 0.059 |  |
| Metothrexate | 0 (0%) | 0 (0%) | 0 (0%) |  |  |
| Steroids | 7 (15%) | 7 (26%) | 0 (0%) | 0.014 |  |
| ECMO support | 13 (27%) | 3 (11%) | 10 (48%) | 0.005 |  |
| RRT | 16 (33%) | 13 (48%) | 3 (14%) | 0.014 |  |
| SAPS II score | 54 (48, 58) | 54 (49, 63) | 54 (42, 58) | 0.4 |  |
| SOFA score | 10 (8, 12) | 10 (8, 12) | 8 (8, 10) | 0.2 |  |
| ^1^n (%); Median (IQR), ^2^Pearson's Chi-squared test; Fisher's exact test, Wilcoxon rank sum test. | | | | | |

Abbreviations: VAP: Ventilator-Associated Pneumonia, COVID-19: Coronavirus Disease 2019, BMI: Body Mass Index, CKD: Chronic Kidney Disease, ECMO: Extracorporeal Membrane Oxygenation, RRT: Renal Replacement Therapy, SAPS II score: Simplified Acute Physiology Score II, SOFA score: Sequential Organ Failure Assessment score.

S7. General characteristics of the population positive to difficult to treat (DTR) gram-negative bacteria during the first episode of VAP.

|  | | **Study group** | |  |  |
| --- | --- | --- | --- | --- | --- |
| **Characteristic** | **Overall**, N = 84^1^ | **NON-COVID-19**, N = 32^1^ | **COVID-19**, N = 52^1^ | **p-value**^2^ |  |
| Sex, male | 63 (75%) | 23 (72%) | 40 (77%) | 0.6 |  |
| Age, years | 64 (57, 72) | 64 (55, 75) | 64 (60, 71) | 0.6 |  |
| BMI, kg/m2 | 27 (24, 31) | 25 (23, 29) | 28 (25, 31) | 0.036 |  |
| Cardiovascular comorbidities | 23 (27%) | 11 (34%) | 12 (23%) | 0.3 |  |
| Diabetes type II | 17 (20%) | 3 (9.4%) | 14 (27%) | 0.052 |  |
| CKD | 11 (13%) | 8 (25%) | 3 (5.8%) | 0.018 |  |
| Respiratory comorbidities | 13 (15%) | 6 (19%) | 7 (13%) | 0.5 |  |
| Alcohol or drug abusers | 2 (2.4%) | 1 (3.1%) | 1 (2.0%) | >0.9 |  |
| Cirrhosis | 1 (1.2%) | 1 (3.1%) | 0 (0%) | 0.4 |  |
| Immunodepression | 8 (9.5%) | 4 (13%) | 4 (7.7%) | 0.5 |  |
| Immunosuppressive therapy | 11 (13%) | 7 (22%) | 4 (7.7%) | 0.094 |  |
| Cyclosporin | 4 (4.8%) | 4 (13%) | 0 (0%) | 0.019 |  |
| Tacrolimus/Everolimus | 0 (0%) | 0 (0%) | 0 (0%) |  |  |
| Azathioprine | 1 (1.2%) | 1 (3.1%) | 0 (0%) | 0.4 |  |
| Mofetyl Mycophenolate | 4 (4.8%) | 4 (13%) | 0 (0%) | 0.019 |  |
| Metothrexate | 1 (1.2%) | 0 (0%) | 1 (1.9%) | >0.9 |  |
| Steroids | 8 (9.5%) | 7 (22%) | 1 (1.9%) | 0.004 |  |
| ECMO support | 24 (29%) | 7 (22%) | 17 (33%) | 0.3 |  |
| RRT | 21 (25%) | 16 (50%) | 5 (9.6%) | <0.001 |  |
| SAPS II score | 50 (39, 57) | 53 (44, 60) | 48 (35, 55) | 0.086 |  |
| SOFA score | 10 (8, 12) | 11 (9, 13) | 8 (7, 11) | 0.005 |  |
| ^1^n (%); Median (IQR), ^2^Pearson's Chi-squared test; Fisher's exact test, Wilcoxon rank sum test. | | | | | |

Abbreviations: VAP: Ventilator-Associated Pneumonia, COVID-19: Coronavirus Disease 2019, BMI: Body Mass Index, CKD: Chronic Kidney Disease, ECMO: Extracorporeal Membrane Oxygenation, RRT: Renal Replacement Therapy, SAPS II score: Simplified Acute Physiology Score II, SOFA score: Sequential Organ Failure Assessment score.

S8. General characteristics of the population positive to multi-drug resistant organisms (MDROs) during the first episode of VAP.

|  | | **Study group** | |  |
| --- | --- | --- | --- | --- |
| **Characteristic** | **Overall**, N = 150^1^ | **NON-COVID-19,** N = 59^1^ | **COVID-19**, N = 91^1^ | **p-value**^2^ |
| Sex, male | 110 (73%) | 42 (71%) | 68 (75%) | 0.6 |
| Age, years | 64 (56, 73) | 67 (56, 77) | 64 (57, 71) | 0.064 |
| BMI, kg/m2 | 27 (24, 31) | 25 (23, 29) | 28 (26, 31) | <0.001 |
| Cardiovascular comorbidities | 40 (27%) | 22 (37%) | 18 (20%) | 0.018 |
| Diabetes type II | 29 (19%) | 8 (14%) | 21 (23%) | 0.15 |
| CKD | 22 (15%) | 17 (29%) | 5 (5.5%) | <0.001 |
| Respiratory comorbidities | 21 (14%) | 9 (15%) | 12 (13%) | 0.7 |
| Alcohol or drug abusers | 3 (2.0%) | 1 (1.7%) | 2 (2.2%) | >0.9 |
| Cirrhosis | 2 (1.3%) | 2 (3.4%) | 0 (0%) | 0.2 |
| Immunodepression | 11 (7.3%) | 6 (10%) | 5 (5.5%) | 0.3 |
| Immunosuppressive therapy | 19 (13%) | 14 (24%) | 5 (5.5%) | 0.001 |
| Cyclosporin | 7 (4.7%) | 7 (12%) | 0 (0%) | 0.001 |
| Tacrolimus/Everolimus | 1 (0.7%) | 1 (1.7%) | 0 (0%) | 0.4 |
| Azathioprine | 2 (1.3%) | 2 (3.4%) | 0 (0%) | 0.2 |
| Mofetyl Mycophenolate | 9 (6.0%) | 9 (15%) | 0 (0%) | <0.001 |
| Metothrexate | 1 (0.7%) | 0 (0%) | 1 (1.1%) | >0.9 |
| Steroids | 15 (10%) | 14 (24%) | 1 (1.1%) | <0.001 |
| ECMO support | 41 (27%) | 12 (20%) | 29 (32%) | 0.12 |
| RRT | 36 (24%) | 23 (39%) | 13 (14%) | <0.001 |
| SAPS II score | 51 (41, 57) | 52 (42, 62) | 48 (41, 56) | 0.2 |
| SOFA score | 10 (8, 12) | 10 (8, 12) | 9 (7, 11) | 0.012 |
| \| \| ^1^n (%); Median (IQR), ^2^Pearson's Chi-squared test; Fisher's exact test, Wilcoxon rank sum test. \| \| --- \| \| \| --- \| --- \|   Abbreviations: VAP: Ventilator-Associated Pneumonia, COVID-19: Coronavirus Disease 2019, BMI: Body Mass Index, CKD: Chronic Kidney Disease, ECMO: Extracorporeal Membrane Oxygenation, RRT: Renal Replacement Therapy, SAPS II score: Simplified Acute Physiology Score II, SOFA score: Sequential Organ Failure Assessment score. | | | | |

S9. General characteristics among survivors and non survivors in VAP population.

|  | | **Survivors vs non-survivors** | |  |
| --- | --- | --- | --- | --- |
| **Characteristic** | **Overall**, N = 201^1^ | **No**, N =125^1^ | **Yes**, N = 76^1^ | **p-value**^2^ |
| Hospital Outcome | 125 (62%) | 125 (100%) | 0 (0%) | <0.001 |
| Sex, male | 147 (73%) | 97 (78%) | 50 (66%) | 0.067 |
| Age, years | 64 (55, 73) | 64 (57, 73) | 65 (53, 74) | 0.5 |
| BMI, kg/m2 | 27 (24, 31) | 27 (24, 31) | 27 (24, 31) | 0.7 |
| Cardiovascular comorbidities | 49 (24%) | 34 (27%) | 15 (20%) | 0.2 |
| Diabetes type II | 35 (17%) | 27 (22%) | 8 (11%) | 0.045 |
| CKD | 27 (13%) | 18 (14%) | 9 (12%) | 0.6 |
| Respiratory comorbidities | 26 (13%) | 16 (13%) | 10 (13%) | >0.9 |
| Alcohol or drug abusers | 7 (3.5%) | 3 (2.4%) | 4 (5.3%) | 0.4 |
| Cirrhosis | 3 (1.5%) | 1 (0.8%) | 2 (2.6%) | 0.6 |
| Immunodepression | 16 (8.0%) | 9 (7.2%) | 7 (9.2%) | 0.6 |
| Immunosuppressive therapy | 24 (12%) | 12 (9.6%) | 12 (16%) | 0.2 |
| Cyclosporin | 7 (3.5%) | 2 (1.6%) | 5 (6.6%) | 0.11 |
| Tacrolimus/Everolimus | 3 (1.5%) | 1 (0.8%) | 2 (2.6%) | 0.6 |
| Azathioprine | 3 (1.5%) | 3 (2.4%) | 0 (0%) | 0.3 |
| Mofetyl Mycophenolate | 8 (4.0%) | 2 (1.6%) | 6 (7.9%) | 0.055 |
| Metothrexate | 1 (0.5%) | 0 (0%) | 1 (1.3%) | 0.4 |
| Steroids | 18 (9.0%) | 10 (8.0%) | 8 (11%) | 0.5 |
| ECMO support | 55 (27%) | 39 (31%) | 16 (21%) | 0.12 |
| RRT | 46 (23%) | 34 (27%) | 12 (16%) | 0.062 |
| SAPS II score | 52 (41, 58) | 54 (44, 59) | 47 (39, 57) | 0.059 |
| SOFA score | 10 (8, 12) | 10 (8, 12) | 8 (7, 11) | 0.033 |
| ^1^n (%); Median (IQR), ^2^Pearson's Chi-squared test; Fisher's exact test, Wilcoxon rank sum test. | | | | |

Abbreviations: VAP: Ventilator-Associated Pneumonia, COVID-19: Coronavirus Disease 2019, BMI: Body Mass Index, CKD: Chronic Kidney Disease, ECMO: Extracorporeal Membrane Oxygenation, RRT: Renal Replacement Therapy, SAPS II score: Simplified Acute Physiology Score II, SOFA score: Sequential Organ Failure Assessment score.

S10. General characteristics of pathogens in the first episode of ventilator acquired pneumonia among dialysis and non-dialysis patients.

|  | | **Dialysis group** | |
| --- | --- | --- | --- |
| **Characteristic** | **Overall**, N = 203^1^ | **No**, N = 155^1^ | **Yes**, N = 48^1^ |
| Number of pathogens determining VAP |  |  |  |
| 0 | 11 (5%) | 5 (3%) | 6 (13%) |
| 1 | 139 (68%) | 113 (73%) | 26 (54%) |
| 2 | 49 (24%) | 34 (22%) | 15 (31%) |
| 3 | 4 (2%) | 3 (2%) | 1 (2%) |
| Gram-negative related VAP | 167 (82%) | 130 (84%) | 37 (77%) |
| Gram-positive related VAP | 45 (22%) | 33 (21%) | 12 (25%) |
| MDR-related VAP | 150 (74%) | 114 (74%) | 36 (75%) |
| XDR-related VAP | 94 (46%) | 71 (46%) | 23 (48%) |
| PDR-related VAP | 3 (1%) | 3 (2%) | 0 (0%) |
| DTR-related VAP | 84 (41%) | 63 (41%) | 21 (44%) |
| ESBL-related VAP | 19 (9%) | 11 (7%) | 8 (17%) |
| Cephalosporins resistance | 122 (60%) | 94 (61%) | 28 (58%) |
| Betalactams resistance | 174 (86%) | 133 (86%) | 41 (85%) |
| Carbapenem resistance | 106 (52%) | 82 (53%) | 24 (50%) |
| Fluoroquinolones resistance | 125 (62%) | 94 (61%) | 31 (65%) |
| Colistin resistance | 20 (10%) | 12 (8%) | 8 (17%) |
| CR-Ab | 55 (27%) | 48 (31%) | 7 (15%) |
| KPC | 48 (24%) | 32 (21%) | 16 (33%) |
| MRSA | 12 (6%) | 6 (4%) | 6 (13%) |
| Cephalosporins resistance | 122 (60%) | 94 (61%) | 28 (58%) |
| Betalactams resistance | 174 (86%) | 133 (86%) | 41 (85%) |
| Carbapenem resistance | 106 (52%) | 82 (53%) | 24 (50%) |
| Fluoroquinolones resistance | 125 (62%) | 94 (61%) | 31 (65%) |
| Colistin resistance | 20 (10%) | 12 (8%) | 8 (17%) |
| ICU length of stay, days | 26 (16, 39) | 26 (17, 37) | 26 (14, 49) |
| Hospital length of stay, days | 30 (22, 60) | 30 (22, 51) | 34 (18, 83) |
| Duration of MV, days | 19 (11, 31) | 18 (12, 28) | 21 (11, 39) |
| ICU mortality | 114 (56%) | 84 (54%) | 30 (64%) |
| Hospital mortality | 125 (62%) | 91 (59%) | 34 (74%) |
| ^1^n (%) | | | |

S11. General characteristics of pathogens in the first episode of ventilator acquired pneumonia among ECMO and non ECMO patients.

|  | | **ECMO group** | |
| --- | --- | --- | --- |
| **Characteristic** | **Overall**, N = 203^1^ | **No**, N = 147^1^ | **Yes**, N = 56^1^ |
| Number of pathogens determining VAP |  |  |  |
| 0 | 11 (5%) | 6 (4%) | 5 (9%) |
| 1 | 139 (68%) | 97 (66%) | 42 (75%) |
| 2 | 49 (24%) | 40 (27%) | 9 (16%) |
| 3 | 4 (2%) | 4 (3%) | 0 (0%) |
| Gram-negative related VAP | 167 (82%) | 124 (84%) | 43 (77%) |
| Gram-positive related VAP | 45 (22%) | 33 (22%) | 12 (21%) |
| MDR-related VAP | 150 (74%) | 109 (74%) | 41 (73%) |
| XDR-related VAP | 94 (46%) | 72 (49%) | 22 (39%) |
| PDR-related VAP | 3 (1%) | 2 (1%) | 1 (2%) |
| DTR-related VAP | 84 (41%) | 60 (41%) | 24 (43%) |
| ESBL-related VAP | 19 (9%) | 18 (12%) | 1 (2%) |
| Cephalosporins resistance | 122 (60%) | 93 (63%) | 29 (52%) |
| Betalactams resistance | 174 (86%) | 126 (86%) | 48 (86%) |
| Carbapenem resistance | 106 (52%) | 77 (52%) | 29 (52%) |
| Fluoroquinolones resistance | 125 (62%) | 92 (63%) | 33 (59%) |
| Colistin resistance | 20 (10%) | 15 (10%) | 5 (9%) |
| CR-Ab | 55 (27%) | 39 (27%) | 16 (29%) |
| KPC | 48 (24%) | 35 (24%) | 13 (23%) |
| MRSA | 12 (6%) | 8 (5%) | 4 (7%) |
| Cephalosporins resistance | 122 (60%) | 93 (63%) | 29 (52%) |
| Betalactams resistance | 174 (86%) | 126 (86%) | 48 (86%) |
| Carbapenem resistance | 106 (52%) | 77 (52%) | 29 (52%) |
| Fluoroquinolones resistance | 125 (62%) | 92 (63%) | 33 (59%) |
| Colistin resistance | 20 (10%) | 15 (10%) | 5 (9%) |
| ICU length of stay, days | 26 (16, 39) | 21 (15, 32) | 32 (26, 46) |
| Hospital length of stay, days | 30 (22, 60) | 29 (19, 60) | 34 (26, 56) |
| Duration of MV, days | 19 (11, 31) | 16 (10, 28) | 25 (16, 37) |
| ICU mortality | 114 (56%) | 77 (53%) | 37 (66%) |
| Hospital mortality | 125 (62%) | 86 (59%) | 39 (71%) |
| ^1^n (%) | | | |
